# Editorial: Methods and applications in respiratory physiology

**DOI:** 10.3389/fphys.2022.1039039

**Published:** 2022-10-04

**Authors:** Walter Araujo Zin

**Affiliations:** Carlos Chagas Filho Institute of Biophysics, Universidade Federal do Rio de Janeiro, Rio de Janeiro, Brazil

**Keywords:** respiratory physiology, pathophysiology, methods, lung, cell, organelle

The Research Topic *Methods and Applications in Respiratory Physiology* focus on various methods and techniques applied to Respiratory Physiology. *Methods and Applications in Respiratory Physiology* welcomed contributions on new or existing methods and protocols that look at the respiratory system under organ, tissue, cellular, subcellular, and molecular lenses. The networking within the system and with other organs and functions of the organism was also highly appreciated. The overall type of contributions encompassed: (i) Methods: Including either existing methods that are significantly improved or adapted for specific purposes or new methods, which may also include primary (original) data; (ii) Protocols: Should provide a detailed description, with pitfalls and troubleshooting, and be of immediate use to the readers. The protocols must be proven to work. (iii) Perspective or General Commentaries on methods and protocols relevant for physiology research; and (iv) Reviews and mini-reviews of current methods and protocols highlighting the important future directions of the field. Hence, contributions based on biological, biochemical, biophysical, engineering, mathematical, behavioral and clinical approaches were encouraged. As a natural result, this Research Topic encompasses interesting topics from the whole lung down to the subcellular level, from biology to mathematics and physics, from models to new devices (Agrawal et al.; Aymerich et al.; Caldeira et al.; Gattarello et al.; Prisk; Krause-Sorio et al.; Sarabia-Vallejos et al.; Alapati and Shaffer; Barbas; Bayat et al.; Biselli et al.; Cao et al.; Choi et al.; Demoulin et al.; Godbole et al.; Guérin et al.; Kim et al.; Miserocchi et al.; Nof et al.; Patel et al.).

Aymerich et al. present a portable, low-cost and specific device for maximal inspiratory and expiratory pressures that could greatly facilitate monitoring of patients at point-of-care sites. Detailed technical information to easily reproduce the device is freely released and its design is available according to the open-source hardware approach. Considering that many lung models can be found in the literature, and pathophysiology and interactions between lungs and ventilators present challenges for modeling efforts not yet thoroughly solved, Agrawal et al. developed a damaged-informed lung ventilator model relying in mathematizing ventilator pressure and volume waveforms. Their model has enough flexibility to reproduce commonly observed variability in clinical (human) and laboratory (mouse) waveform data, providing high fidelity estimates of pulmonary pathophysiological conditions. Demoulin et al. introduce a physical analog to assess surgical face mask airflow resistance during tidal ventilation. The physical analog was made of a plaster cast dummy head connected through a pneumotachograph to a series of bellows inflated/deflated by a ventilator. They measured the added respiratory mechanical load due to ten surgical masks tested in four different ways. Nof et al. describe a multicompartmental human airway on-chip platform to serve as a preclinical *in vitro* benchmark underlining regional lung crosstalk for viral infections pathways. The platform mimics key elements of the respiratory system including nasal passages that serve as the alleged origin of infections, the mid-bronchial airway region, and the peripheric acinar zone. The authors share detailed methodologies for fabricating, assembling, calibrating, and using the platform, including open-source fabrication files.

Sarabia-Vallejos et al. combine micro computed-tomography (micro-CT) and computational geometry algorithms to evaluate the regional distribution of key morphological parameters throughout the whole rat lung. They found that regional porosity, alveolar surface density and surface-to-volume ratio present a uniform distribution in normal lungs that is unaffected by gravitational effects. They also introduce a new dehydration protocol including methanol-PBS solution before dehydration that avoids the sample shrinking commonly found in ethanol-based protocols. Synchrotron radiation imaging methods are clearly described by Bayat et al. It offers unique properties of coherence, employed in phase-contrast imaging, and high flux as well as a wide energy spectrum. These properties allow the quantitative determination of lung morphology, and map regional lung ventilation, perfusion, inflammation, aerosol particle distribution and biomechanical properties with microscopic spatial resolution. Barbas discusses the use of thoracic-computed tomography (CT) in the assessment and treatment of patients with acute respiratory distress syndrome (ARDS) and COVID-19 pulmonary disease. Other than helping to correctly diagnose ARDS, CT can assist the adjustment of positive end-expiratory pressure, ideal tidal volume, and to position the patient during invasive mechanical ventilation. CT has been the most sensitive imaging technique to pinpoint pulmonary involvement in COVID-19 patients. Choi et al. applied CT image matching to assess the degree of pulmonary motion in idiopathic interstitial disease such as interstitial pneumonia (UIP) e nonspecific interstitial pneumonia (NUIP) patients. They report that lung motion quantified by image registration-based lower lobe dorsal-basal displacement may be used to assess the degree of motion reflecting limited motion owing to fibrosis in UIP and NUIP subjects.

Gattarello et al. studied pigs mechanically ventilated for 48 h and divided them into two groups: high or low pleural pressure. They assessed respiratory mechanics, hemodynamics, fluid, sodium, and osmotic balance at specific time points along the experimental duration. As a conclusion, the mechanical power and pleural pressure were positively associated with hemodynamic support maneuvers, increased sodium and fluid retention, and pulmonary edema. Interestingly, Miserocchi et al. report that the tendency to develop lung edema in edemagenic conditions, i.e., work in the face of hypoxia, is directly proportional to the ratio of lung capillary blood volume to the diffusion capacity of the alveolar membrane, as suggested by an estimate of the mechanical properties of the respiratory system with the forced oscillation technique. Cao et al. searched three online electronic databases, yielding seven studies, and report that elevated central venous pressure (CVP) and brain natriuretic peptide (BNP) levels are associated with extubation failure in critically ill patients and further suggest that BNP levels are more valuable than CVP levels in predicting extubation outcomes.

Efficiency of pulmonary gas exchange has long been assessed using the alveolar-arterial difference in partial pressure of oxygen (A-aDO_2_). However, this measurement is invasive and unsuitable for serial measurements, since it requires arterial blood sample(s). According to Prisk, recent technological advances provide for portable and rapidly responding measurements of PO_2_ and PCO_2_ in expired gas, which combined with the usual determination of arterial oxygen saturation *via* pulse oximetry (SpO_2_) make practical a non-invasive surrogate measurement of A-aDO_2_. In fact, the approach shares the underlying basis of the measurement of gas exchange efficiency and simplifies the determination of the oxygen deficit. Godbole et al. advance that lung resection surgery carries significant risks of postoperative pulmonary complications (PPC). The authors prospectively determined the utility of resting measurements of physiologic dead space (VC) and physiologic dead space to tidal volume ratio (VD/VT) in predicting PPC in patients who underwent robotic-assisted lung resection and found that preoperative resting VD was significantly elevated in patients with PCC. They advance that the increase in resting VD may be a potentially useful predictor of PCC in patients under similar conditions.

Inspiratory muscle training (IMT) may improve respiratory and cardiovascular functions in obstructive sleep apnea (OSA). However, the available IMT protocols cannot be completed by some OSA patients. Hence, Krause-Sorio et al. describe a new 13-week OSA-friendly protocol for IMT and applied it to five sedentary OSA patients. The practice and subsequent 65% IMT resistance targets resulted in inspiratory strength gains that reached a steady-state by the end of 10 weeks of training and no report of adverse effects. Patel et al. present an extensive review concerning *methods and applications in respiratory physiology* and pathophysiology of neuromuscular and chest wall disorders. The authors cover respiratory muscles, reduction in respiratory resistance and elastance, obesity, respiratory mechanical loads and reactional compensation, dyspnea, muscle fatigue, cough, respiratory muscle failure, evaluation of respiratory muscle function, airflow limitation, control of ventilation, sleep quality, oximetry and capnography, gene therapy, electrical and magnetic muscle stimulation, respiratory mechanics in neuro muscular diseases. Additionally, they present future promising research fields. Guérin et al. describe pathophysiological aspects of ARDS such as impairment of lung microvasculature, loss of alveolar aeration as a result of lesions to the small peripheral airways, as evidenced by high-resolution imaging techniques, atelectrauma, expiratory flow limitation, pattern of airway opening pressure disclosed in the inspiratory volume-pressure curve, functional interplay between airway opening pressure and expiratory flow limitation, and individualization of PEEP settings. Another review article by Biselli et al. addresses the use of respiratory mechanics in basic science to investigate asthma and chronic obstructive pulmonary disease (COPD), discusses the use of lung mechanics in clinical care and its role in the development of mechanical ventilators. Finally, they explore some or the difficult questions that intensive care personnel still face when managing respiratory failure.

The assessment of mitochondrial function in organs and tissues is essential to better understand their biochemistry, physiology, and pathophysiology. The evaluation of mitochondrial function is usually accomplished in isolated mitochondria, permeabilized fibers, or cells. These techniques are very well-defined in several types of tissue, e.g., heart, kidney, liver, adipose tissue, and brain. On the other hand, assessment of lung mitochondrial function presents difficulties associated with obtaining isolated, intact, coupled, and functional mitochondria. The methodological difficulty of obtaining viable lung mitochondria derives mainly from an elevated fatty acid content, low number of mitochondria in the cell, fibrous and air-filled tissue, and the required amount of tissue. To solve this issue, Caldeira et al. present an isolation protocol specific for lung tissue mitochondria and detail the mitochondrial function pertaining to several respiratory complexes.

Electrical impedance tomography (EIT) is an evolving technique that monitors physiological functions based on temporal changes in electrical conductivity in different tissues. Kim et al. measured tidal volumes by EIT in rabbit pups and compared the measurements to those provided by a mechanical ventilator. Three groups of animals were used: untreated (preterm), surfactant-treated (preterm) and control (term puppies). In all instances the results provided by EIT and mechanical ventilator were not different.

In a review article, Alapati and Shaffer address the use of inert liquids for respiratory support and as a vehicle to deliver biological agents to the respiratory system. They cover the respiratory support with inert liquids, clinical and non-clinical studies using inert liquids, and drug/gene product administration.

In conclusion, the studies published in this Research Topic confirm the broad range of methods and techniques used to address respiratory function in health and disease.

